# Right pneumothorax with congenital pericardial defect showed right atrium mimicking bulla in surgery

**DOI:** 10.1186/s40792-022-01457-y

**Published:** 2022-05-26

**Authors:** Yasoo Sugiura, Toshinori Hashizume

**Affiliations:** Department of General Thoracic Surgery, National Hospital Organization Kanagawa National Hospital, 666-1 Ochiai Hadano, Kanagawa, 257-8585 Japan

**Keywords:** Pericardial defect, Pneumothorax, Thoracic surgery, Pleuropericardium window

## Abstract

**Background:**

Congenital pericardial defect (CPD) is found incidentally in cases of pneumothorax. CPD is seen in left side rather than right side and it is not generally known among thoracic surgeons how the inside of the pericardial space can be seen from the thoracic cavity in cases of pericardial defect.

**Case presentation:**

A 52-year-old man with dyspnea was referred to our hospital because of the diagnosis of right pneumothorax. Chest radiography showed a right lung collapse and a pneumopericardium on the left side. Despite insertion of a chest tube, air leakage prolonged, bullectomy at the apex of the right lung was performed under thoracoscopy. During surgery, thoracoscope showed that the right atrium seemed as if it had been a non-pedunculated bulla or cardiac cyst. Heart beating, continuity with the heart, and the absence of respiratory motion could distinguish the right atrium from a bulla, and pericardial defect was confirmed. Preoperatively, the patient had no cardiac symptoms related to the CPD, and therefore, it was determined that a procedure to close the CPD was not necessary. Any complication and recurrence did not occur 6 months after surgery.

**Conclusions:**

Right pneumothorax with CPD showed right atrium mimicking bulla in surgery. It is important to consider correction of CPD if there are cardiac symptoms at the onset of pneumothorax, and not to misinterpret the right atrium as a bulla.

## Background

During thoracic surgery, congenital pericardial defect (CPD) is incidentally found. A reported prevalence of approximately 0.002–0.004% [1]. The most cases of CPD are asymptomatic. However, there is a potential life-threatening risk [2]. CPD is seen in left side rather than right side and it is not generally known among thoracic surgeons how the inside of the pericardial space can be seen from the thoracic cavity in cases of pericardial defects. In the present report of a case of right pneumothorax with congenital pericardial defect, we described how to assess the possibility of cardiac hernia and the surgical findings.

## Case presentation

**Fig. 1:**
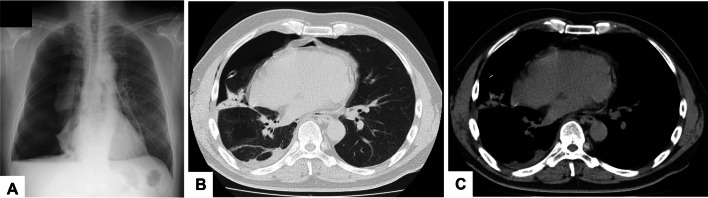
Chest radiography (**A**) shows a right lung collapse and a pneumopericardium on the left side. Computed tomography (CT) before surgery with lung window settings (**B**) and with mediastinal window settings (**C**) shows air in the pericardial sac and the right atrium was prolapsed into the right thoracic cavity

**Fig. 2:**
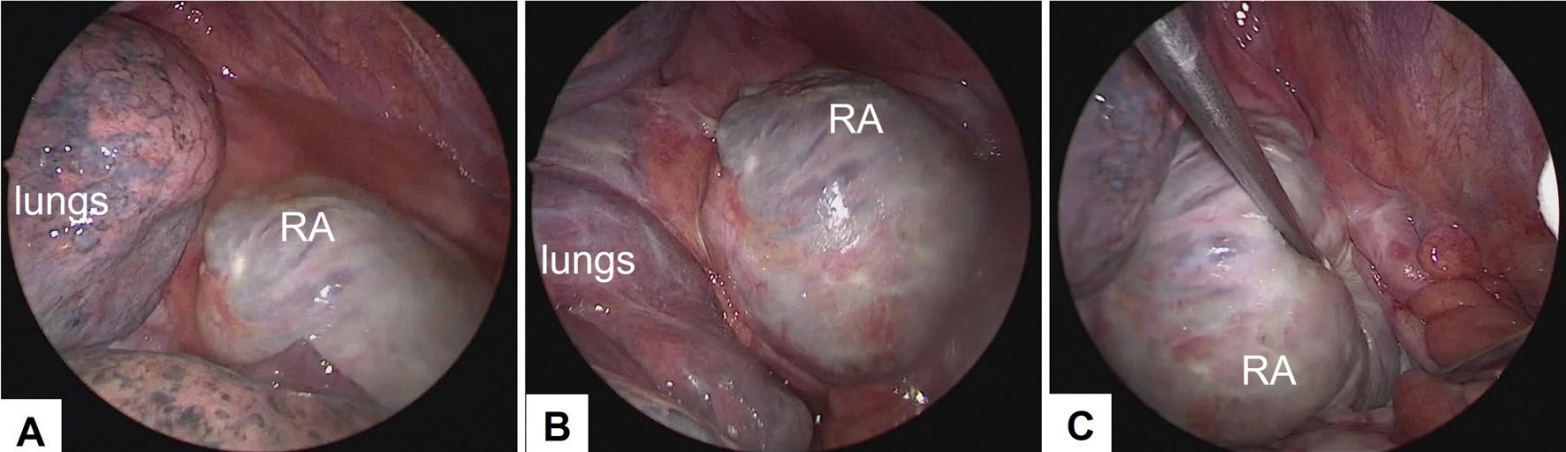
During surgery, the right atrium (RA) seemed as if it had been a non-pedunculated bulla or pericardiac cyst (**A**). When the lungs were moved, heart beat was observed without respiratory motion (**B**). Continuity to the heart was observed (**C**). Heart beating, continuity with the heart, and the absence of respiratory motion could distinguish the right atrium from a bulla, and a pericardial defect was confirmed

**Fig. 3:**
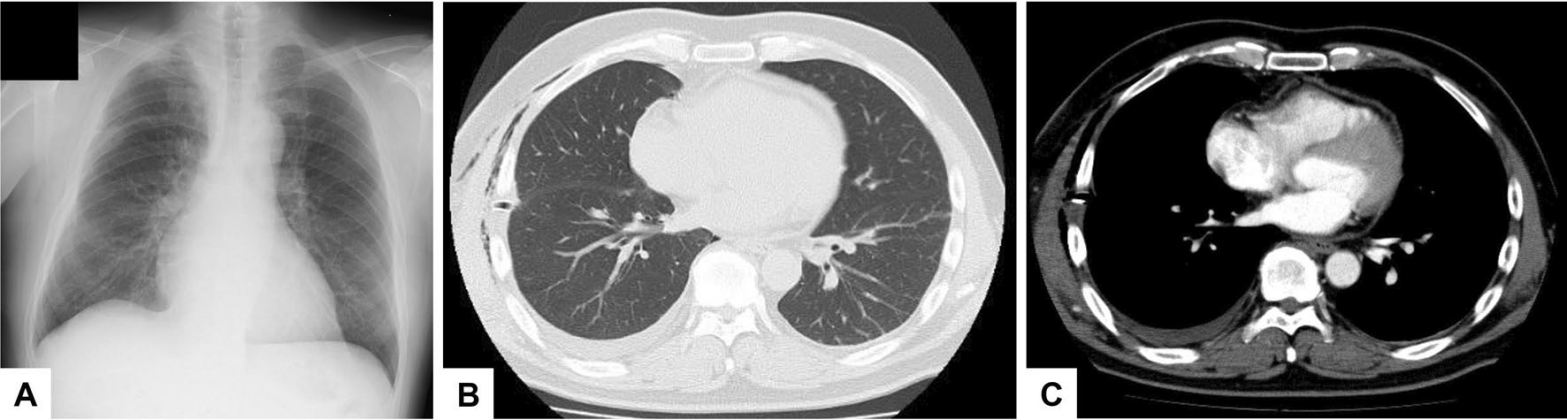
On chest radiography after surgery (**A**), the pneumopericardium seen preoperatively on the left side of the heart had disappeared, and the protruding right atrium on the right side of the heart had returned to the mediastinum. Computed tomography after surgery with lung window settings (**B**) shows that the right atrium was pushed back into the mediastinum by the inflated lung. The enhanced computed tomography after surgery with mediastinal window settings (**C**) shows that the right atrium was present at the site of the pericardial defect, consistent with the surgical findings

A 52-year-old man with dyspnea was referred to our hospital because of the diagnosis of right pneumothorax. He had history of allergic dermatitis and he was a heavy smoker (two packs/day for 32 years). Chest radiography showed a right lung collapse and a pneumopericardium on the left side (Fig. 1A). Computed tomography (CT) showed air in the pericardial sac (Fig. 1B and C). QRS axis of ECG was normal. Despite insertion of chest tube, air leakage prolonged and bullectomy at the apex of the right lung was performed under thoracoscopy. During surgery, the right atrium seemed as if it had been a non-pedunculated bulla or pericardiac cyst (Fig. 2A). Heart beating, continuity with the heart, and the absence of respiratory motion could distinguish the right atrium from a bulla, and pericardial defect was confirmed (Fig. 2B and C). Preoperatively, the patient had no cardiac symptoms related to the CPD, and therefore, it was determined that a procedure to close the CPD was not necessary. Compared to the CT before and after surgery, it showed that the right atrium was prolapsed into the right thoracic cavity when the lung was collapsed (Fig. 1B and C), and the right atrium was pushed back into the mediastinum by the inflated lung after surgery (Fig. 3A–C). After surgery, cardiac ultrasound echo did not reveal any abnormalities. Any complication and recurrence of pneumothorax did not occur 6 months after surgery.

## Discussion

CPD is a developmental defect that results from faulty partitioning of the pleuropericardiac cavity during the 5th week of development [3]. There were 257 surgical cases of pneumothorax in our hospital between March 2010 and December 2021, and two cases including the present case had pleural defects [4]. The frequency was 0.8%. It has been reported that complications are less likely to occur when the pericardial defect is wider [5]. In cases where the pericardial defect is narrow, there have been reports of myocardial infarction, arrhythmia, angina pectoris, syncope, and sudden death [6–10]. A search using PubMed for case reports of pneumothorax with CPD resulted in 8 cases [4, 11–17]. Nine cases, including the present case, are listed in Table 1. Eight of the nine cases had left-sided CPD. This was the first case of right-sided CPD. Symptoms at the onset of pneumothorax were dyspnea in 6 cases and chest pain in 4 cases. Palpitations were present in only one case [11]. In ECG, axial deviation was noted in two cases and abnormal Q waves in one case. Surgical treatment for pericardial defects was performed in only one case, because the transient attacks of chest pain and palpitation which were brought on when she lay on her left side and were promptly relieved by a change of position [11]. In other cases, no hemodynamic abnormalities were happened. These indicate that the CPD should be corrected if there are cardiac symptoms that improve with a change of position, while correction of the CPD is not necessary if there are no cardiac symptoms.

**Table 1:** Cases of pneumothorax with congenital pericardial defect

Author	Year	Age	Sex	Side	Symptoms	ECG	Surgical intervention
Kostiainen	1975	38	F	Left	Chest pain, palpitation dyspnea	ND	+
Nakagawa	1985	17	M	Left	Dyspnea, cough	Normal	−
Pickhardt	1998	39	M	Left	Chest pain, dyspnea	Left-axis deviation (− 55°)	−
Sugiyama	2015	18	M	Left	ND	Abnormal Q wave on leads V1 and V2	–
Sugiura	2016	67	M	Left	Chest pain	Tachycardia, normal axis	−
Murasawa	2016	72	M	Left	Dyspnea	Sinus, right-axis deviation	−
Date	2020	16	M	Left	Chest pain, dyspnea	Right-axis deviation within the normal range	−
Loo	2021	22	M	Left	Shortness of breath	ND	−
Sugiura	2022	52	M	Right	Dyspnea	Normal axis	−

*ND* not described

CT images before and after lung inflation showed that the heart was pushed back into the mediastinum by the inflated lung in the present case and the previous report [4]. These findings support that the inflated lung buttresses the mediastinum structure.

In the present report, we experienced a case pneumothorax involved with CPD on the right side. The case involving right pneumothorax and pericardial defect had a deviation of the heart. The surgical findings showed that the right atrium looked as if it was a non-pedunculated bulla or pericardiac cyst. The heartbeat, continuity with the heart, and respiratory motion made us confident that it was the right atrium. In most of the cases of pneumothorax involving CPD, no specific treatment is required once the lung re-expands and the heart returns to its proper position, because there are no major cardiovascular events until the pneumothorax occurs in adulthood [18].

## Conclusion

This case report illustrates two important points. It is important to consider correction of CPD if there are cardiac symptoms at the onset of pneumothorax, and not to misinterpret the right atrium as a bulla during surgery.

## Data Availability

There are no available data and materials to be shared.

## Abbreviations

CPD: Congenital pericardial defect
CT: Computed tomography
MRI: Magnetic resonance imaging
ECG: Electrocardiogram
